# Myeloid cell expressed proprotein convertase FURIN attenuates inflammation

**DOI:** 10.18632/oncotarget.11106

**Published:** 2016-08-05

**Authors:** Zuzet Martinez Cordova, Anna Grönholm, Ville Kytölä, Valentina Taverniti, Sanna Hämäläinen, Saara Aittomäki, Wilhelmiina Niininen, Ilkka Junttila, Antti Ylipää, Matti Nykter, Marko Pesu

**Affiliations:** ^1^ Team Immunoregulation, Institute of Biosciences and Medical Technology (BioMediTech), University of Tampere, Tampere, Finland; ^2^ Team Computational Biology, Institute of Biosciences and Medical Technology (BioMediTech), University of Tampere, Tampere, Finland; ^3^ Department of Food, Environmental and Nutritional Sciences (DeFENS), Division of Food Microbiology and Bioprocessing, Universita degli Studi di Milano, Milan, Italy; ^4^ School of Medicine, University of Tampere, Tampere, Finland; ^5^ Fimlab Laboratories, Pirkanmaa Hospital District, Tampere, Finland; ^6^ Department of Dermatology, Tampere University Hospital, Tampere, Finland

**Keywords:** cytokine, FURIN, LysM, macrophage, TGF-β1, Immunology and Microbiology Section, Immune response, Immunity

## Abstract

The proprotein convertase enzyme FURIN processes immature pro-proteins into functional end- products. FURIN is upregulated in activated immune cells and it regulates T-cell dependent peripheral tolerance and the Th1/Th2 balance. FURIN also promotes the infectivity of pathogens by activating bacterial toxins and by processing viral proteins. Here, we evaluated the role of FURIN in LysM+ myeloid cells *in vivo*. Mice with a conditional deletion of FURIN in their myeloid cells (LysMCre-*fur*^(fl/fl)^) were healthy and showed unchanged proportions of neutrophils and macrophages. Instead, LysMCre-*fur*^(fl/fl)^ mice had elevated serum IL-1β levels and reduced numbers of splenocytes. An LPS injection resulted in accelerated mortality, elevated serum pro-inflammatory cytokines and upregulated numbers of pro-inflammatory macrophages. A genome-wide gene expression analysis revealed the overexpression of several pro-inflammatory genes in resting FURIN-deficient macrophages. Moreover, FURIN inhibited *Nos2* and promoted the expression of *Arg1*, which implies that FURIN regulates the M1/M2-type macrophage balance. FURIN was required for the normal production of the bioactive TGF-β1 cytokine, but it inhibited the maturation of the inflammation-provoking TACE and Caspase-1 enzymes. In conclusion, FURIN has an anti-inflammatory function in LysM+ myeloid cells *in vivo*.

## INTRODUCTION

The components of the innate immunity implement the recognition and elimination of microbes through a complex machinery involving factors that require a post-translational proteolytic activation step to perform their effector functions. The functional maturation of dormant pro-proteins is catalyzed by the proprotein convertase subtilisin/kexin (PCSK) enzymes (PCSK1-2, FURIN, PCSK5-7, membrane-bound transcription factor site 1, PCSK9) [1]. The first seven PCSKs operate at a similar target site consisting of basic amino acids [K/R)-(X)n-(K/R)↓, with *n* being 0, 2, 4 or 6 and X any amino acid], which results in a significant degree of functional redundancy in target recognition and processing *in vitro*. However, humans bearing mutations in the PCSK genes as well as PCSK-deficient experimental animal models have gene-specific phenotypes, which argues for substrate specificity *in vivo* [2].

FURIN was the first discovered and is thus the most studied conventional PCSK enzyme [3]. FURIN is ubiquitously expressed, and in cells it catalyzes the maturation of its targets in the secretory pathway, endosomes and on the cell surface. Due to its widespread expression, FURIN has a plethora of reported targets including cytokines, chemokines and growth factors as well as other proteases like matrix metalloproteinases. In mice, the expression of *Furin* is essential for embryonic development, which has imposed limitations to our understanding of its cell-type specific function *in vivo* [3, 4]. However, the phenotypes of tissue-specific *Furin* deficient mice have demonstrated that FURIN cannot be compensated for by other PCSK enzymes in endothelial cells (Tie2Cre) or in T lymphocytes (CD4Cre) [5, 6].

FURIN's regulatory role is also implicated in multiple human pathologies. For example, FURIN processes the beta-secretase enzyme in Alzheimer's disease, SNPs in the *Furin* gene are associated with blood pressure levels, and elevated FURIN expression promotes metastatic activity in various cancer types, and the protein is found in advanced atherosclerotic plaques [7–10]. FURIN is an important modulator of the T-cell-dependent adaptive immunity; it becomes upregulated by T-cell-receptor-mediated signaling and through the IL-12/STAT4 pathway in T helper type 1 cells [11, 12]. A conditional deletion of FURIN in T cells results in the aberrant polarization of T helper cells, a lack of a protective cell-mediated host-defense as well as the spontaneous development of autoimmunity in aging animals due to a breakage in peripheral CD4+Foxp3+ T-regulatory-cell-dependent immune tolerance [6, 13]. Consequently, targeting the activity of FURIN/PCSK has been reported to be beneficial for the experimental treatment of, for example, malignancies and rheumatoid arthritis [9, 14–16].

Previous reports have also implicated a role for PCSK enzymes in spleen [17]. FURIN is expressed in splenic red pulp, a zone enriched with macrophages, which regulate extramedullary myelopoiesis, the removal of senescent red cells, the cross-presentation of antigens as well as tolerance to self-antigens [18–20]. In addition, PCSK1 deficient mice show a marked disorganization of the marginal zone and red pulp [21]. In addition to the cellular pro-proteins also the components of several infectious agents, including the envelopes of the HI and Influenza viruses as well as the toxins of *Bacillus antracii* and *Pseudomonas aeruginosa* require a PCSK-dependent proteolysis step to exert their pathogenic function [22–24]. Therefore, inhibitors can protect the host from invading PCSK-dependent pathogens and serve as adjuvants to antibiotics [25]. As the innate immune system forms the first line of defense, targeting PCSKs specifically in myeloid cells could be a potent and well-tolerated strategy to block infections. However, the consequences of PCSK inhibition specifically in the cells of the innate immune system *in vivo* have remained ambiguous. To address this conundrum we have here characterized a novel tissue-specific knock-out mouse model, in which FURIN is deleted in Lysozyme M positive cells, i.e. chiefly in activated macrophages and granulocytes (LysMCre-*fur*^(fl/fl)^) [26].

## RESULTS AND DISCUSSION

### LysMCre-*fur*^(fl/fl)^ mice have a reduced number of splenocytes and elevated levels of the pro-inflammatory IL-1β cytokine in their serum

**Figure 1:**
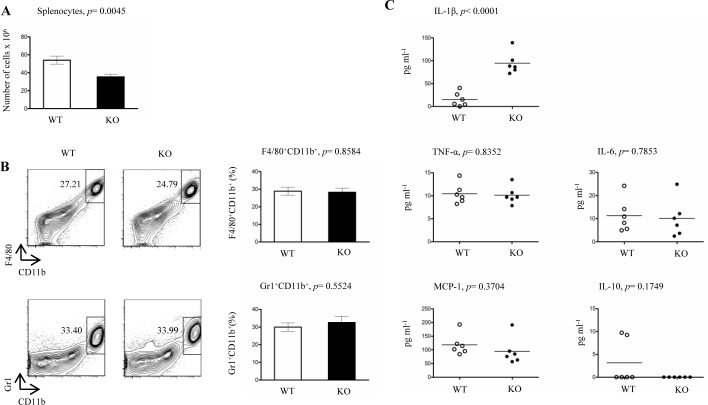
Proportions of splenocytes, peritoneal macrophages, neutrophils and serum cytokines in steady state LysMCre-*fur***^fl/fl^** mice **A.** Total numbers of live splenocytes from LysMCre-*fur*^(fl/fl)^ (KO) and WT littermate control mice were calculated using trypan blue staining (*n* = 7/genotype, 6-8 weeks of age). **B.** Flow cytometric analyses of F4/80^+^CD11b^+^ (macrophages) and Gr1^+^CD11b^+^ (neutrophils) peritoneal cells were performed from LysMCre-*fur*^(fl/fl)^ and WT littermate control mice (*n* = 4/genotype, 6-8 weeks of age). **C.** Levels of serum cytokines from steady state LysMCre-*fur*^(fl/fl)^ and WT littermate control mice (*n* = 6/genotype, 6-8 weeks old mice). Plots represent average ± SEM. Statistics were calculated with the two-tailed unpaired Student's *t*-test.

Previous studies using germ-line deletions and siRNA have demonstrated that PCSK1 and PCSK7 modulate the secretion process of innate cytokines [21] and rescue unstable MHCI molecules on dendritic cells, respectively [27]. Others and we have further shown that FURIN is upregulated in the LPS activated CD14+ cells [10], and in the plasma of chronic typhoid carriers [28]. To directly address whether FURIN expression in myeloid cells regulates immunity *in vivo*, we generated a mouse model with a conditional deletion of FURIN in LysM+ cells. LysMCre-*fur*^(fl/fl)^ mice were born under normal Mendelian ratios, and unlike T-cell specific CD4Cre-*fur*^(fl/fl)^ mice [6], they did not show age-related health problems such as inflammatory bowel disease (supplementary Figure S1A, B). Notably, in contrast to the reportedly large spleens of PCSK1 KO mice [21] LysMCre-*fur*^(fl/fl)^ animals had significantly reduced numbers of splenocytes compared to controls, which could indicate that FURIN regulates hematopoiesis in LysM+ cells, or cellular homeostasis in the spleen (Figure 1A, supplementary Figure S1C, D). The mechanism(s) leading to the reduction in splenocyte numbers in LysMCre-*fur*^(fl/fl)^ animals remains unclear, but the lowered FURIN levels in the bone marrow (ca. 60% less mRNA expression, supplementary Figure S2A) could disrupt the delicate microenvironment by reducing the proteolytic maturation of hematopoietic growth factors or extracellular matrix receptors [29].

As expected, FURIN was almost completely (>95% less mRNA) absent from both the neutrophils and macrophages of LysMCre-*fur*^(fl/fl)^ mice (supplementary Figure S2B, C). A flow cytometric analysis demonstrated that the lack of FURIN did not significantly alter the proportions of macrophages (F4/80^+^CD11b^+^) and neutrophils (Gr1^+^CD11b^+^) in the peritoneal cavity (Figure 1B), spleen (supplementary Figure S3A) or bone marrow (supplementary Figure S4A). We next analyzed whether deleting FURIN in myeloid cells imposed secondary effects on lymphoid cells, and found that the gross numbers of splenic CD3^+^ T and B220^+^ B cells were unaffected (supplementary Figure S3B). Instead, there was a small, but significant reduction in the numbers of CD3^+^CD4^+^CD8^−^ T helper cells in the spleen while the proportion of CD3^+^CD4^−^CD8^+^ cytotoxic T cells was elevated (supplementary Figure S3C). The lower ratio of splenic CD4^+^/CD8^+^ T cells has previously been associated with inflammation [30], but the quantification of CD62L^+^ T cells did not reveal marked differences in the numbers of memory T cells between LysMCre-*fur*^(fl/fl)^ and littermate control animals (supplementary Figure S3C). Collectively, the analyses of different cell compartments indicate that FURIN expression in LysM+ cells is not essential for the development of neutrophils or macrophages, but it promotes the development of splenocytes and may also indirectly affect the CD4^+^/CD8^+^ T cell ratio in the spleen.

We have previously demonstrated that deleting FURIN in T cells results in the upregulation of both the Th1 and Th2 signature cytokines in serum [6]. To address how FURIN expression in LysM+ cells controls the production of innate cytokines in steady state we measured the levels of pro-inflammatory IL-1β, TNF-α, IL-6 and MCP-1 cytokines as well as the anti-inflammatory IL-10 in serum (Figure 1C). These data demonstrated that FURIN expression in myeloid cells attenuates the production of pro-inflammatory IL-1β, which is indicative of an auto-inflammatory phenotype [31, 32].

### Genome-wide RNA microarray analysis reveals the upregulation of several pro-inflammatory genes in FURIN deficient macrophages

Although proprotein convertases process their target molecules post-translationally, deleting PCSKs also results in aberrant gene expression signatures, which gives clues about the mechanism of the actions of PCSKs in a given cell type [6, 12, 33]. To address how FURIN regulates the genetic signature in macrophages we profiled their gene expression using qRT-PCR and microarray analyses. Similarly to human CD14+ monocytes [10] FURIN was efficiently upregulated in mouse peritoneal macrophages that were activated with LPS+/− IFNγ *in vitro*, (supplementary Figure S2B). The lack of FURIN in endothelial cells (Tie2Cre) causes the compensatory expression of other biochemically redundant PCSKs [5], which interferes with the interpretation of FURIN's biological significance. However, we observed that deleting FURIN in macrophages did not affect the expression of other PCSK enzymes (supplementary Figure S2D). These data indicate that the LysMCre-mediated deletion can be reliably used to assess the specific role of FURIN in LysM+ cells.

**Figure 2:**
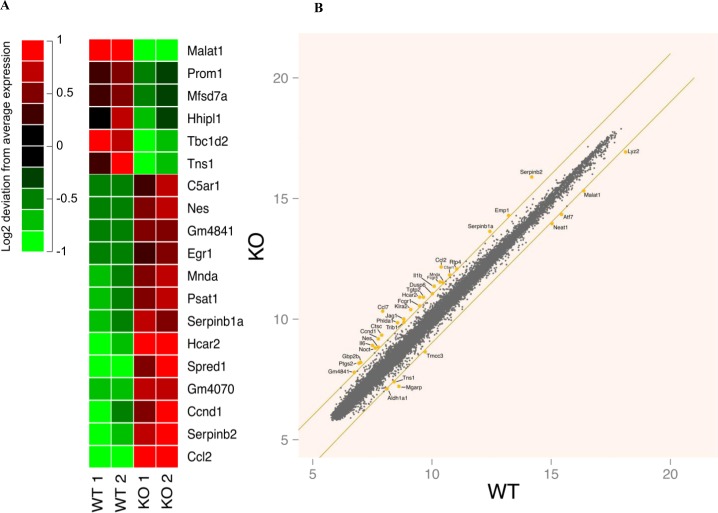
Genome-wide gene expression analyses in resting wild type and FURIN deficient peritoneal macrophages **A.** The 19 most differentially (log fold change > |1| and multiple testing corrected *p*-value < 0.05) expressed genes between wild type and FURIN deficient unstimulated peritoneal macrophages. For visualization purposes, the mean of each gene's expression has been subtracted from individual expression values to highlight the differences between the samples. The red and green colors indicate an induction or suppression of gene expression, respectively, relative to the mean across samples. The figure shows two biological replicates for both genotypes. **B.** A scatter plot of the mean gene expression across replicates of wild type and FURIN deficient unstimulated peritoneal macrophages. Selected genes with a log fold change > |1| have been highlighted. The orange lines show the limit where the log fold change equals 1 or −1.

In order to characterize the FURIN-dependent global gene expression patterns we performed a genome-wide microarray analysis using resting peritoneal macrophages from LysMCre-fur^(fl/fl)^ and littermate WT animals (Figure 2A, 2B). FURIN deficient peritoneal macrophages displayed a reproducible upregulation of many genes expressed in activated macrophages, such as *Serpinb1a, Serpinb2, Hcar2, Egr1, Il6, Il1β, Ptgs2, Ccl2, Ccl7 and C5ar1* [34–38]. In addition, we observed an enhanced expression of *Dusp6* and *Fcgr1,* which are downregulated in alternatively activated macrophages [39]. In contrast, among the downregulated genes of FURIN deficient macrophages, we detected for example *Atf7* whose deletion is associated with the constitutive activation of macrophages [40]. FURIN is induced by its substrate TGF-β1 [41]. Accordingly, the expression of *Ccnd1* was enhanced in the absence of FURIN, similarly to what was seen in TGF-β1 null cells [42]. These data suggest that FURIN has an intrinsic inhibitory function on the expression of genes that associate with the activation of pro-inflammatory M1 type macrophages [43].

### LysMCre-*fur*^(fl/fl)^ mice show increased lethality, upregulated pro-inflammatory cytokines and elevated numbers of Ly6-C+ macrophages after an LPS injection

**Figure 3:**
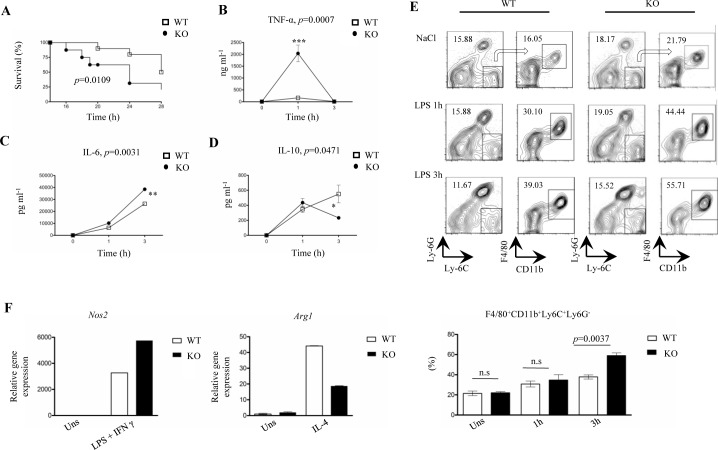
LPS-challenged LysMCre-*fur***^fl/fl^** mice exhibit higher mortality, accelerated inflammation and an upregulated pro-inflammatory macrophage response **A.** Kaplan Meier survival curve for LysMCre-*fur*^(fl/fl)^ and WT littermate control mice (*n* = 8/genotype, 6-8 weeks of age, all male) after the intraperitoneal injection of a single lethal dose of LPS (25 mg/kg). LysMCre-*fur*^(fl/fl)^ mice exhibited a significantly higher mortality than WT littermate controls during the first 24 h. **B.**-**D.** Level of serum cytokines TNF-α, IL-6, IL-10 in LysMCre-*fur*^(fl/fl)^ and WT littermate controls mice (*n* = 4/genotype, 6-8 weeks of age) after an intraperitoneal injection with NaCl (0.9%) or 100 μg/kg of LPS (plots represent average ± SEM). Statistics were calculated with the two-tailed unpaired Student's *t*-test. **E.** Flow cytometric analysis of a splenic F4/80^+^CD11b^+^Ly6C^+^Ly6G^−^ cell population in LysMCre-*fur*^(fl/fl)^ mice and WT littermate control mice (*n* = 3/genotype, 6-8 weeks of age) injected with NaCl (0.9%) or LPS (100 μg/kg) at 0, 1h and 3h. The arrows indicate the gating strategy. (Plots represent average ± SEM). Statistics were calculated using the two-tailed unpaired Student's *t*-test. **F.** *Nos2* and *Arg1* mRNA expression was assessed by quantitative RT-PCR in wild type and FURIN deficient peritoneal macrophages treated with LPS+IFN-γ (1 μg/ml+20 ng/ml) or IL-4 (50 ng/ml). One representative experiment out of three independent experiments is shown.

Both the high IL-1β levels in serum and the results from the microarray analysis pointed to a pro-inflammatory phenotype in LysMCre-*fur*^(fl/fl)^ mice. To test if FURIN in LysM+ cells also restrains inflammatory responses *in vivo* we subjected mice to lipopolysaccharide (LPS) triggered inflammation. LysMCre-*fur*^(fl/fl)^ and littermate WT mice were first challenged with a single i.p. injection of 25 mg/kg (LPS) and monitored for 72 hours (Figure 3A). We observed a significantly higher mortality in LysMCre-*fur*^(fl/fl)^ mice 24 hours post-injection (62% mortality in KO *vs*. 38% mortality in WT) (*p* = 0.0109), which indicates an increased sensitivity to a pro-inflammatory stimulus.

A lower dose of LPS (100 μg/kg) was then used to evaluate how the myeloid-cell-expressed FURIN regulates cytokine levels in the serum and the generation/migration of pro-inflammatory F4/80^+^CD11b^+^Ly6C^+^Ly6G^−^ monocytes/macrophages, which are characterized by accelerated inflammatory effector functions including the high production of IL-6, IL-1β, TNF-α, NOS2 and CCL2 [44, 45]. LPS-injected LysMCre-*fur*^(fl/fl)^ mice had significantly enhanced levels of the pro-inflammatory cytokines TNF-α and IL-6 1 and 3 hours post-infection, respectively, whereas the production of anti-inflammatory IL-10 was reduced at the 3-hour time-point (Figure 3B-3D).

In addition, at 3 hours post-injection there was a significant augmentation in the proportion of F4/80^+^CD11b^+^Ly6C^+^Ly6G^−^ M1 type macrophages in the spleen (Figure 3E). A qRT-PCR analysis on *ex vivo* activated FURIN KO macrophages further showed an upregulation in the expression of *Nos2* (M1 marker gene) upon an LPS/IFN-γ stimulus, whereas the IL-4 induced expression of *Arg1* (M2 marker gene) was clearly reduced [43] (Figure 3F). Previously, the prominence of the M1 over the M2 macrophage phenotype has been associated with an increased susceptibility to a septic shock [46, 47], as was observed in LPS-challenged LysMCre-*fur*^(fl/fl)^ mice.

**Figure 4:**
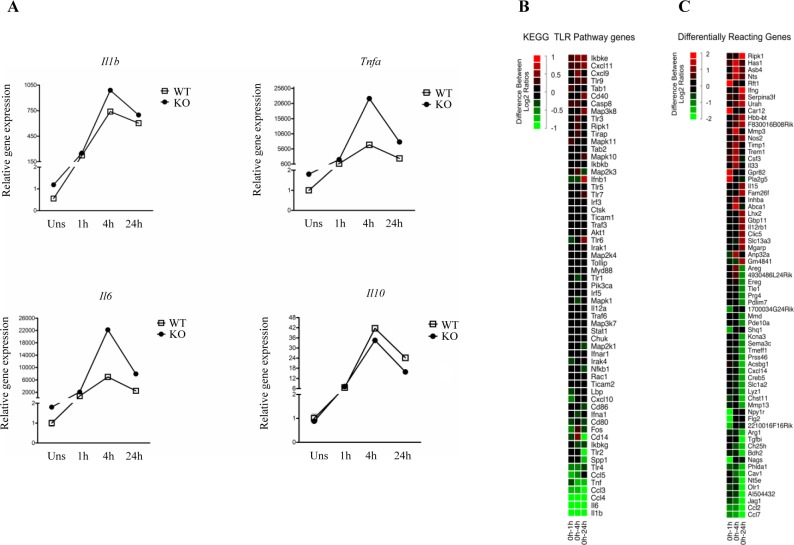
LPS-induced gene expression dynamics in wild type and FURIN deficient peritoneal macrophages **A.** mRNA expression levels were determined using quantitative RT-PCR, and the normalized expression in the unstimulated WT sample was arbitrarily set to 1. Curves show changes in the expression of *Il1b*, *Tnfa*, *Il6* and *Il10* mRNAs in WT and FURIN KO peritoneal macrophages that were left unstimulated or were stimulated with LPS (1μg/ml) for 1-24h as indicated. The housekeeping gene 18S was used to normalize the gene expression. (The figure shows one representative experiment of two independent experiments ± SEM). **B.** Microarray analysis of the TLR signaling pathway genes. Log2 fold changes for all genes in both WT and FURIN KO peritoneal macrophages were computed for each time point with an LPS (1μg/ml) stimulation, respective to the unstimulated condition. At each time point, the ratio of the fold changes illustrates the degree of difference in the LPS response over time. The figure shows the average of two independent experiments. The red color indicates an increase and the green a decrease in the log2 fold change difference. **C.** Genome wide analysis of the genes with the strongest differential response to a LPS stimulation between WT and FURIN KO peritoneal macrophages. A selection of differentially responding genes showing greatest differences between ratios at one or several time points are presented. Data were processed as in B.

PCSKs control the quantity and activation of the human Toll-like receptor 7 (TLR7) host responses by direct proteolysis [48]. We next tested if the expression levels of LPS/TLR4-dependent genes are affected by the FURIN deficiency in peritoneal macrophages. First, the time course changes in the mRNA expression of the *Il1b, Tnfa*, *Il6* and *Il10* genes indicated an upregulated base line expression but roughly similar dynamics in response to LPS in FURIN deficient macrophages compared to controls (Figure 4A). Likewise, a focused analysis of TLR-associated mRNAs using microarray data showed only minimal differences between the dynamic expression patterns of WT and FURIN KO macrophages (Figure 4B). However, the dynamic expression of several other pro-inflammatory genes, including *Trem1, Nos2, Il15, Il33, and Il12rb1*, was inhibited by FURIN in LPS activated macrophages (Figure 4C) [49–52]. These findings were accompanied by the repression of the genes *Ch25h, Olr1 and Arg1*, which are typically induced in alternatively activated macrophages [38]. Finally, we evaluated the expression of pro- and anti-inflammatory cytokines in peritoneal macrophages that were stimulated with various TLR ligands and IFN-γ (supplementary Figure S5). These results show that the anti-inflammatory function of FURIN cannot be attributed to a specific stimulus, but can be seen in TLR2, TLR4 and TLR7/8 activated macrophages. Collectively, these data suggest that FURIN is dispensable for the immediate TLR4 responses, but underscore the importance of FURIN as an anti-inflammatory factor in both resting and activated macrophages.

### FURIN deficient macrophages secrete less bioactive TGFβ-1 cytokine but show elevated expression levels of the activated TNF-α Converting Enzyme (TACE) and Caspase-1 p20

The production of several cytokines secreted by macrophages, including TNF-α, IL-1β and TGF-β1, is dependent on proteolytic processing in the cell [53]. Specifically, TGF-β1 is initially produced as an inactive pro-cytokine, which is converted into an active factor via a complex post-translational cascade involving a cleavage step catalyzed by FURIN [6, 54]. Further, undermined TGF-β1 signaling has been associated with an impaired transit between the macrophage (M1/M2) phenotypes, sustained inflammation and delayed wound healing [55]. Also, TNF-α Converting Enzyme (TACE), which releases soluble TNF-α from its membrane-bound precursor, is proteolytically activated by a FURIN-like proprotein convertase [56] whose deletion results in the development of an anti-inflammatory phenotype in macrophages [57]. In addition, the activation/deactivation of the Caspase-1 cascade, which directly processes IL-1β, plays an important role in the dynamics of macrophage polarization [58]. Previous data also imply a functional connection between PCSK activity and IL-1β; the proteolytic cleavage of the anthrax toxin by FURIN activates Caspase-1 in macrophages [59] and high levels of IL-1β have been observed in an experimental model of arthritis in mice that were treated with a FURIN inhibitor [60].

**Figure 5:**
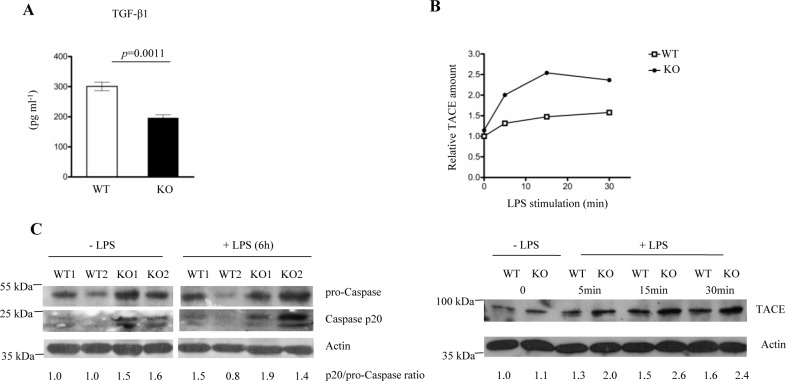
FURIN deficient macrophages produce less bioactive TGF-β1 and show hyperactivation of TACE and Caspase-1 p20 **A.** The production of bioactive TGF-β1 was measured in the supernatants of unstimulated wild type and FURIN deficient peritoneal macrophages using an enzyme linked immunosorbent assay (ELISA) (*n* = 3/genotype). Plots represent average ± SEM. Statistics were calculated with the two-tailed unpaired Student's *t*-test. **B.** WT and FURIN deficient peritoneal macrophages were left unstimulated or were stimulated with LPS (1 μg/ml) for 5-30 minutes as indicated. Mature Tumor Necrosis Factor-α-Converting Enzyme (TACE) (93 kDa) and β-Actin (43 kDa) were detected by western blotting. Normalized (TACE/Actin) levels are presented in the upper panel. Shown is one representative experiment out of three replicates with similar results. **C.** FURIN deficient or littermate wild type bone marrow macrophages (*n* = 2/genotype) were left unstimulated or were stimulated with ultrapure LPS (500 ng/ml) for 2 hours, then ATP (1mM) was added to the cells for 15 min. Pro-Caspase (55 kDa), Caspase-1 p20 (20 kDa) and actin (43 kDa) were detected with western blotting.

In order to find out if FURIN regulates the proteolytic maturation of the aforementioned inflammatory cytokines in macrophages we analyzed the secretion of bioactive TGF-β1 as well as the proteolytic activation of TACE and Caspase-1. First, FURIN KO macrophages were found to secrete significantly lower levels of bioactive TGF-β1 in ELISA analyses than cells from wild-type controls, whereas the *Tgfb1* mRNA levels were not affected (Figure 5A, supplementary Figure S6). In contrast, we found that the lack of FURIN upregulated the mature TACE protein in LPS-stimulated peritoneal macrophages (Figure 5B), which is in line with the elevated TNF-α production in LPS-challenged LysMCre-*fur*^(fl/fl)^ mice *in vivo* (Figure 3B). These data also indicate that FURIN is not the bona-fide PCSK that activates TACE, and suggest that another macrophage-expressed and LPS-induced PCSK enzyme, such as PCSK6 or PCSK7 (supplementary Figure S2D), could be more important for the maturation of TACE *in vivo* [61]. Finally, an analysis of Caspase-1 processing also showed a higher production of Caspase-1p20 in FURIN deficient macrophages (Figure 5C). These data are consistent with the observed higher levels of serum IL-1β and reveal a novel proteolytic regulator (FURIN) for this key pro-inflammatory cytokine.

The analyses of proteolytically activated macrophage cytokines thus showed that a FURIN deficiency reduces the production of anti-inflammatory TGF-β1 but upregulates the activation of the TACE and Caspase-1 enzymes. These findings together with the inherent upregulation of pro-inflammatory mRNAs collectively contribute to the pro-inflammatory phenotype of LysMCre-*fur*^(fl/fl)^ mice. Understanding the underlying mechanism(s) of aberrant cytokine expressions by FURIN deficient macrophages clearly requires further studies, but at least reduced TGFβ-1 maturation is likely to play a role [62]. In conclusion, our data indicate that inhibiting FURIN specifically in innate immune cells could strengthen host responses. This, together with the plausible reduction in the activation of pathogens, could be beneficial for treating and preventing PCSK-dependent infections.

## MATERIALS AND METHODS

### Experimental animals

Mice bearing floxed *fur* alleles [4] were backcrossed six times with C57BL/6 mice. LysMCre mice with the C57BL/6 background were purchased from Taconic. LysMCre mice were bred with *fur*^(fl/fl)^ animals to generate myeloid-specific FURIN knockout mice LysMCre-*fur*^(fl/fl)^. Mice were housed under pathogen-free standard conditions. All mouse experiments were performed in accordance with the National Animal Experiment Board, Finland, (Permit# ESAVI/2837/04.10.07/2015).

### Isolation, culture and *ex vivo* activation of macrophages from the mouse peritoneal cavity and of neutrophils from the bone marrow

Peritoneal cells were extracted from the peritoneal cavity and cultured following a previously published protocol [63]. After 1 h incubation WT and FURIN deficient peritoneal macrophages were stimulated with LPS (1 μg/ml, *E. coli* 0127:B8 serotype; Sigma-Aldrich, St Louis, MO, USA), TLR ligands (Zymosan: 10 μg/ml, R848: 1 μg/ml; both from InvivoGen, San Diego, CA, USA) and/or cytokines (IFN-γ: 20 ng/ml, IL-4: 50 ng/ml; PeproTech, Rocky Hill, NJ, USA). After stimulation, the cells were scraped to dislodge them and collected for RNA extraction (RNAeasy, Qiagen, Düsseldorf, Germany). Neutrophils were isolated from the bone marrow of LysMCre-*fur*^(fl/fl)^ and wild-type mice with the Anti-Ly-6G MicroBead Kit (Miltenyi Biotec Norden AB, Lund, SE) and were left unstimulated or were stimulated with 100 μM of fMLP for 1 hour at 37°C.

### Flow cytometric analyses

Peritoneal, splenic and bone marrow cells were analyzed using flow cytometry (BD FACSCanto™ II) and the FlowJo software (Treestar Inc, Ashland, OR, USA). Anti-CD16/CD32 (Clone: 93) (eBioscience, San Diego, CA, USA) was used to block Fc receptors and cell populations were surface stained following the eBioscience FACS protocol with PerCP-Cy5.5- labeled anti- F4/80 (BM8), PE-labeled anti-Gr-1 (RB6-8C5), PE-Cy7-labeled anti-CD11b (M1/70), FITC-labeled anti-B220 (RA3-6B2), APC-labeled anti-CD3, PE-Cy7 labeled anti-CD4 (RM4-5), APC-H7 labeled anti-CD8 (53-6.7), PE-labeled anti-CD62L (MEL-14), PE-labeled anti-Ly-6G (RB6-8C5), APC labeled anti-Ly-6C (HK1.4), PE-Cy7-labeled anti-CD11c (N418), FITC-labeled anti-CD11b (M1/70) (eBioscience, San Diego, CA, USA).

### Cytokine measurements

Serum cytokines were measured in both steady state and LPS injected mice. Briefly, LysMCre-*fur*^(fl/fl)^ and WT littermate mice (6-8 weeks of age) were injected intraperitoneally with NaCl (0.9%) or LPS (100 μg/kg, *E. coli* 111:B4 serotype; Sigma-Aldrich) and serum was collected at the 0, 1, 3 h time points. The serum cytokines TNF-α, IL-6, MCP-1 and IL-10 were quantified using the BD™ Cytometry Bead Array Mouse Inflammation Kit (Catalog # 552364) according to the manufacturer's instructions (BD Biosciences, Franklin Lakes, NJ, USA). IL-1β levels were measured with mouse IL-1 beta ELISA Ready-SET-Go!^®^ (eBioscience, San Diego, CA, USA) (Catalog # 88-7013-22). Bioactive TGF-β1 was measured in the supernatants of cultured peritoneal macrophages isolated from LysMCre-*fur*^(fl/fl)^ and WT littermate mice using Human/Mouse TGF beta 1 ELISA Ready-SET-Go! (2nd Generation) (Catalog # 88-8350-76) (eBioscience, San Diego, CA, USA).

### LPS induced endotoxemia

LysMCre-*fur*^(fl/fl)^ and WT littermate mice (6-8 weeks of age) were injected intraperitoneally with a lethal dose of LPS (25 mg/kg, *E. coli* 0111:B4 serotype; Sigma Aldrich, St Louis, MO, USA) or with NaCl (0.9%) as a control. Survival of the mice was monitored for 72 h at 3-hour intervals.

### Quantitative real-time polymerase chain analyses

The mRNA expression in stimulated and non-stimulated peritoneal macrophages was assessed using qRT-PCR. Briefly, total RNA was extracted using the RNeasy kit (Qiagen, Düsseldorf, Germany), quantified using a NanoDrop ND-2000 (Thermo Fisher Scientific, Massachusetts, USA) and transcribed into cDNA with the iScript Select cDNA Synthesis Kit (Bio-Rad, Hercules, CA, USA). Gene expression levels were examined using the Bio-Rad CFX96 Real-Time System and the Sso Fast Eva Green Supermix (Bio-Rad, Hercules, CA, USA). The primers used for quantitative real-time PCR are shown in the supplementary Table S1. Gene expression was normalized to 18s levels; the normalized threshold cycle (Ct) values were subtracted from the target Ct values of each sample (deltaCt). The relative levels of the target mRNA were calculated as 2-deltadeltaCt.

### Microarray data analysis

Two biological replicates of FURIN KO and WT peritoneal macrophages were left unstimulated or were stimulated for 1, 4 and 24 hours with LPS (1 μg/ml)(*E. coli* 0127:B8 serotype; Sigma Aldrich, St Louis, MO, USA). The samples were collected and the RNA was isolated using Qiagen RNeasy on-column DNase (Qiagen, Düsseldorf, Germany). 50 ng of purified RNA was amplified and dye labeled using Agilent's Low Input Quick Amp Labeling kit and RNA Spike In kit (Agilent Technologies, California, USA). Initial and purified RNA contents along with dyed and amplified cRNA contents were measured with a NanoDrop ND-2000 (Thermo Fisher Scientific, Massachusetts, USA). RNA and cRNA quality was inspected using the Agilent 2100 Bioanalyzer RNA 6000 Nano kit (Agilent Technologies, California, USA). 300ng of Cy-3 and 300ng of Cy-5 labeled sample were hybridized together on an Agilent Mouse Chip 8×60K (Design ID 028005) (Agilent Technologies, California, USA) overnight at 65°C using the Gene Expression Hybridization kit. The chips were washed with the Gene Expression Wash Pack according to the instructions. The chips were scanned using an Agilent Technologies Scanner model G2565CA using the scan profile AgilentG3_GX_2Color. Scan results were converted into numerical data by the Agilent Feature Extraction software version 10.7.3. The data was analyzed using the R software [64]. Raw probe level intensity values were normalized with loess regression and quantile normalization using R package “limma” to robustly generate sample-wise comparability [65].

Differential gene expression was evaluated using the log2-transformed fold change difference and statistical testing using the two-sample Student's *t*-test. Genes showing an average expression lower than the log2 transformed intensity value 6 were filtered out as non-informative. The Kyoto Encyclopedia of Genes and Genomes (KEGG) pathway database was used to obtain a gene set involved in Toll-like receptor signaling. [66]. The raw microarray data are available at Gene Expression Omnibus (GEO): http://www.ncbi.nlm.nih.gov/geo/query/acc.cgi?token=ililkmqqvvqlryn&acc=GSE84117.

### Western blot analyses

For the Caspase-1 experiment bone marrow macrophages were cultured using a standard protocol [63] and were left unstimulated or were stimulated for 6 hours with ultrapure LPS (*E. coli* 0111:B4, 500 ng/ml; InvivoGen, San Diego, CA, USA). Cells were washed twice with PBS and incubated with ATP (1mM, Sigma Aldrich, St Louis, MO, USA). Next, the cells were collected and lysed in a buffer specific for Caspase-1 (50 mM Tris, pH 7.4, 150 mM NaCl, 2 mM Ethylenediaminetetraacetic acid (EDTA) pH: 8.2 mM Ethyleneglycoltetraacetic acid (EGTA) pH: 7.5, 10% Glycerol, 1% Triton X-100, 50 mM Sodium fluoride, 200 μM Sodium vanadate) [67]. For the TACE-1 experiment, peritoneal macrophages were left unstimulated or were stimulated with LPS for 5-30 minutes (1 μg/ml, *E. coli* 0127:B8 serotype; Sigma Aldrich, St Louis, MO, USA). Next, the cells were collected and lysed in a buffer specific for TACE (1% Triton X-100, 15 mM NaCl, 50 mM Tris HCL pH 7.4, protease inhibitors cocktail (Roche), 10mM 1,10-phenanthroline) [68]. Equal amounts of proteins were separated on 12% polyacrylamide gels and transferred onto nitrocellulose membranes (Whatman, GE Healthcare, Pollards Wood, UK). The proteins were probed with a primary antibody, either anti-ADAM17-cytoplasmic domain (ab39162; abcam) or anti-caspase-1-t/ICE (AHZ0082; Invitrogen), followed by a HRP-conjugated secondary antibody (RnD systems, Minneapolis, MN, USA). Anti-actin (MAB1501R; Merck Millipore, Billerica, MA, USA) was used as a loading control. Immunoblots were visualized with an ECL detection system (GE Healthcare, Pollards Woods, UK). Signal intensities were analyzed using the NIH ImageJ software.

### Statistical analysis

Statistical analyses were performed using the two-tailed unpaired Student's *t*-test. *P* < 0.05 indicates statistical significance and the variability is depicted using the standard error of the mean (SEM). The Kaplan Meier test was applied to estimate survival in the LPS induced endotoxemia experiment.

## SUPPLEMENTARY MATERIALS



## Conventions and Abbreviations

PCSK: Proprotein Convertase Subtilisin/Kexin type
FBS: Fetal Bovine Serum
ELISA: Enzyme-Linked Immunosorbent Assay
CBA: Cytometric Bead Array
qRT-PCR: quantitative Reverse Transcription Polymerase Chain Reaction
IL: Interleukin
TLR: Toll Like Receptor
LPS: Lipopolysaccharide
R848: Resiquimod
IFN-γ: Interferon gamma
TNF-α: Tumor Necrosis Factor alpha
MCP-1: Monocyte Chemotactic Protein 1
TACE: Tumor Necrosis Factor Alpha Converting Enzyme
ADAM17: ADAM Metallopeptidase Domain 17
*Ptgs2*: Cyclooxygenase-2
CXCL: C-X-C motif Chemokine
CCL: Chemokine (C-C motif) Ligand
*Serpinb1a*: Serine (or cysteine) peptidase inhibitor, clade B, member 1a
*Serpinb2*: Serpin Peptidase Inhibitor, Clade B (Ovalbumin), Member 2
*Hcar2*: Niacin Receptor 1
*Egr1*: Early Growth Response 1
*C5ar*: Complement Component 5a Receptor 1
*Dusp6*: Dual specificity phosphatase 6
*Fcgr1*: Fc Fragment of IgG, High Affinity Ia, Receptor
*Atf7*: Activating Transcription Factor 7
*Ccnd1*: Cyclophilin C
CD11b: Cluster of Differentiation molecule 11b, Integrin alpha M
Ly6C: Lymphocyte antigen 6C
Ly6G: Lymphocyte antigen 6G
*Trem1*: Triggering receptor expressed on myeloid cells 1
*Il12rb1*: Interleukin 12 Receptor, Beta 1
*Ch25h*: Cholesterol 25-Hydroxylase
*Olr1*: Oxidized Low Density Lipoprotein (Lectin-Like) Receptor 1
*Nos2*: inducible Nitric Oxide Synthase 2
*Arg*1: Arginase 1
