# Supplementary material for: Myeloid cell expressed proprotein convertase FURIN attenuates inflammation
Source: Oncotarget. 2016 Aug 5;7(34):54392–404. doi: 10.18632/oncotarget.11106 (PMC5342350; doi:10.18632/oncotarget.11106)
Supplement: Supplementary file 1 [file oncotarget-07-54392-s001.pdf]

Myeloid cell expressed proprotein convertase FURIN attenuates inflammation

Supplementary Material

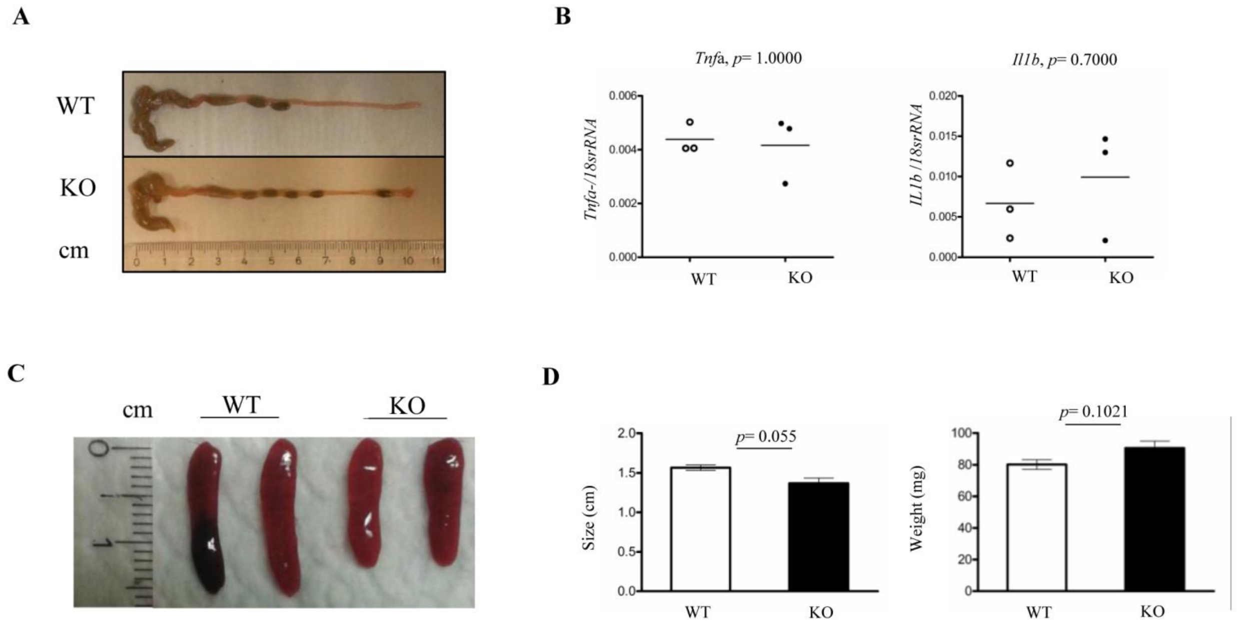

Figure S1 Gut and spleen morphology and cytokine gene expression in the large intestine. A. A representative picture of the gut gross morphology in LysMCre-*fur*$^{(fl/fl)}$ and WT littermate controls mice at 16-19 weeks of age. **B.** mRNA expression was assessed by quantitative RT-PCR to determine the expression of the *Tnfa* and *Il1b* genes in the large intestines of LysMCre-*fur*$^{(fl/fl)}$ and WT littermate control mice, n=3/genotype, 16-19 weeks of age. **C.** A representative picture of the spleen gross morphology in LysMCre-*fur*$^{(fl/fl)}$ and WT littermate controls (n=2+2). **D.** Bar graphs represent size and weight averages ± SEM, n=3/genotype, 6-8 weeks of age. Statistics were calculated using the two-tailed unpaired Student's t-test.

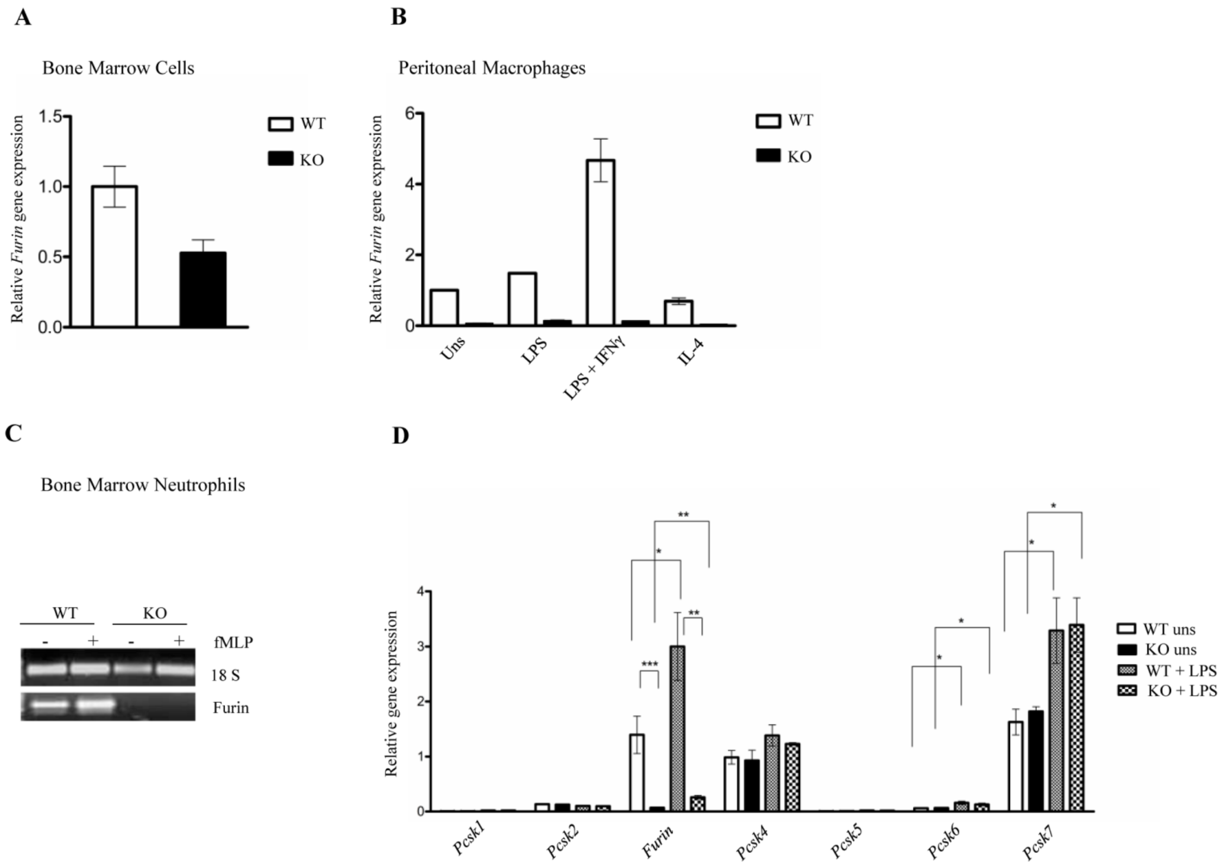

Figure S2 The *Pcsk* gene expressions in LysMCre-*fur*$^{(fl/fl)}$ mice. A. Bone marrow cells were isolated from the femurs and tibias of LysMCre-*fur*$^{(fl/fl)}$ and WT littermate control mice (n=2/genotype, 6-8 weeks of age). *Furin* mRNA expression was assessed by quantitative RT-PCR. Plots represent average ± SEM. **B.** WT and FURIN KO peritoneal macrophages were left unstimulated or were stimulated *in vitro* with LPS (1 µg/ml) and/or cytokines: IFN-γ (20 ng/ml); IL4 (50 ng/ml). *Furin* mRNA expression was assessed by quantitative RT-PCR. The figure shows one representative experiment out of three performed. **C.** Neutrophils were isolated from the bone marrow of LysMCre-*fur*$^{(fl/fl)}$ and WT littermate control mice and were left unstimulated or stimulated with 100 µM of fMLP. *Furin* mRNA expression was assessed by quantitative RT-PCR. The figure shows the gel electrophoresis of *Furin* amplification products. The housekeeping gene 18S was used to normalize the gene expression. **D.** mRNA expressions of the

Pcsk genes were assessed by quantitative RT-PCR in unstimulated and LPS stimulated (1 µg/ml for 4 hours) WT and FURIN KO peritoneal macrophages. The normalized expression of *Furin* in WT samples was set to 1. The housekeeping gene 18S was used to normalize the gene expressions. Statistics were calculated using the two-tailed unpaired Student's t-test. Error bars=SEM (n=4/genotype, *p<0.05, **p<0.01***, p<0.001).

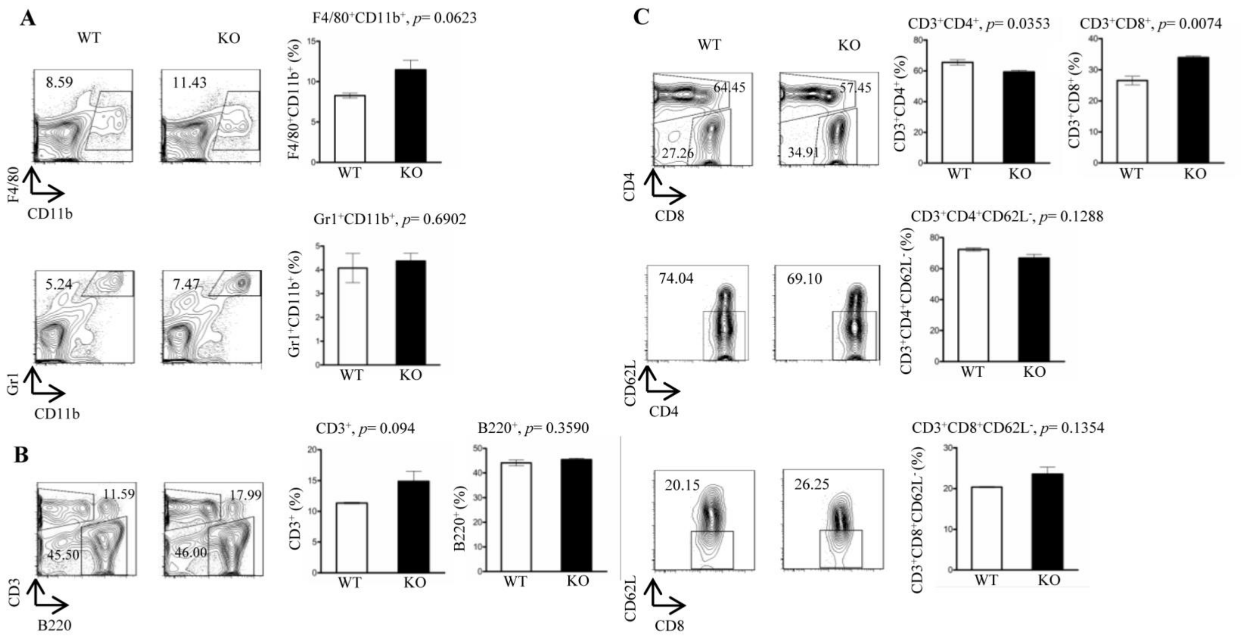

Figure S3. Proportions of immune cell populations in the spleens of LysMCre-*fur*$^{(fl/fl)}$ and littermate wild type mice. A–C. Representative flow cytometry contour plots and plotted mean values of splenic F4/80$^+$CD11b$^+$, Gr1$^+$CD11b$^+$, CD3$^+$, B220$^+$, CD3$^+$CD4$^+$, CD3$^+$CD8$^+$, CD3$^+$CD4$^+$CD62L$^-$ and CD3$^+$CD8$^+$CD62L$^-$ cell populations are shown. (n=4/genotype, 6-8 weeks of age) (Plots represent average ± SEM). Statistics were calculated using the two-tailed unpaired Student's t-test.

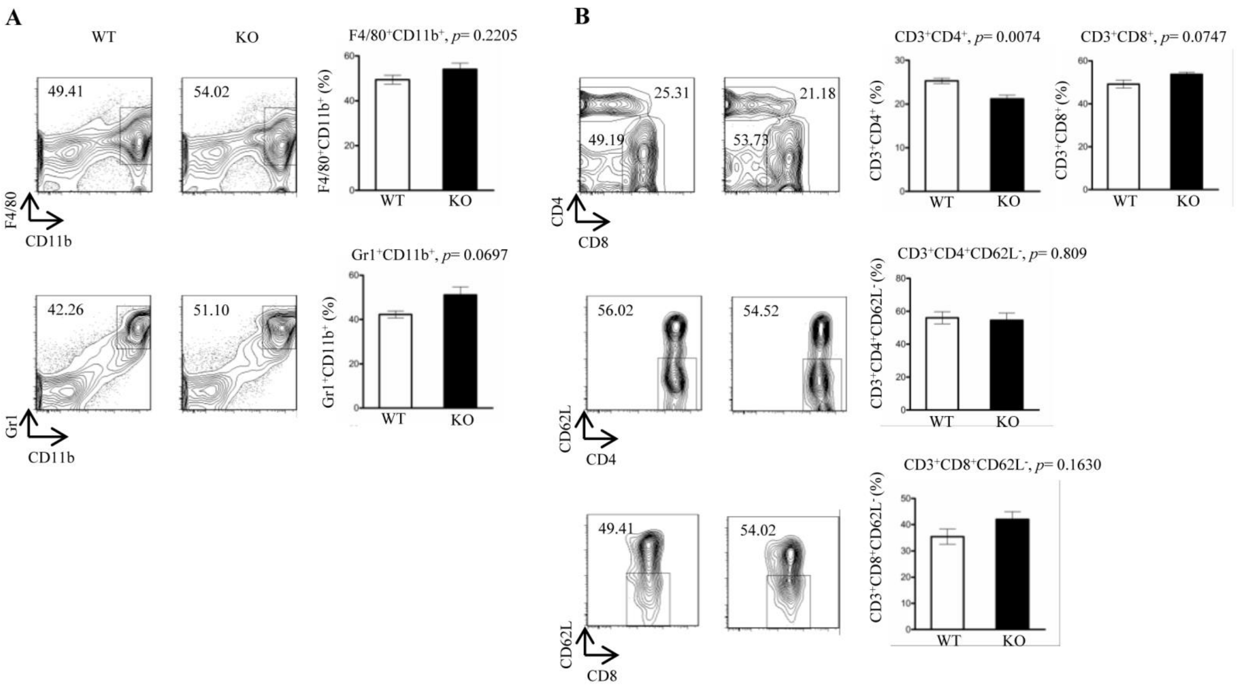

Figure S4. Proportions of immune cell populations in the bone marrow of LysMCre-*fur*$^{(fl/fl)}$ and littermate wild type mice. A-B. Representative flow cytometry contour plots and plotted mean values of bone marrow F4/80$^+$CD11b$^+$, Gr1$^+$CD11b$^+$, CD3$^+$CD4$^+$, CD3$^+$CD8$^+$, CD3$^+$CD4$^+$CD62L$^-$ and CD3$^+$CD8$^+$CD62L$^-$ cell populations are shown. (n=4/genotype, 6-8 weeks of age) (Plots represent average ± SEM). Statistics were calculated using the two-tailed unpaired Student's t-test.

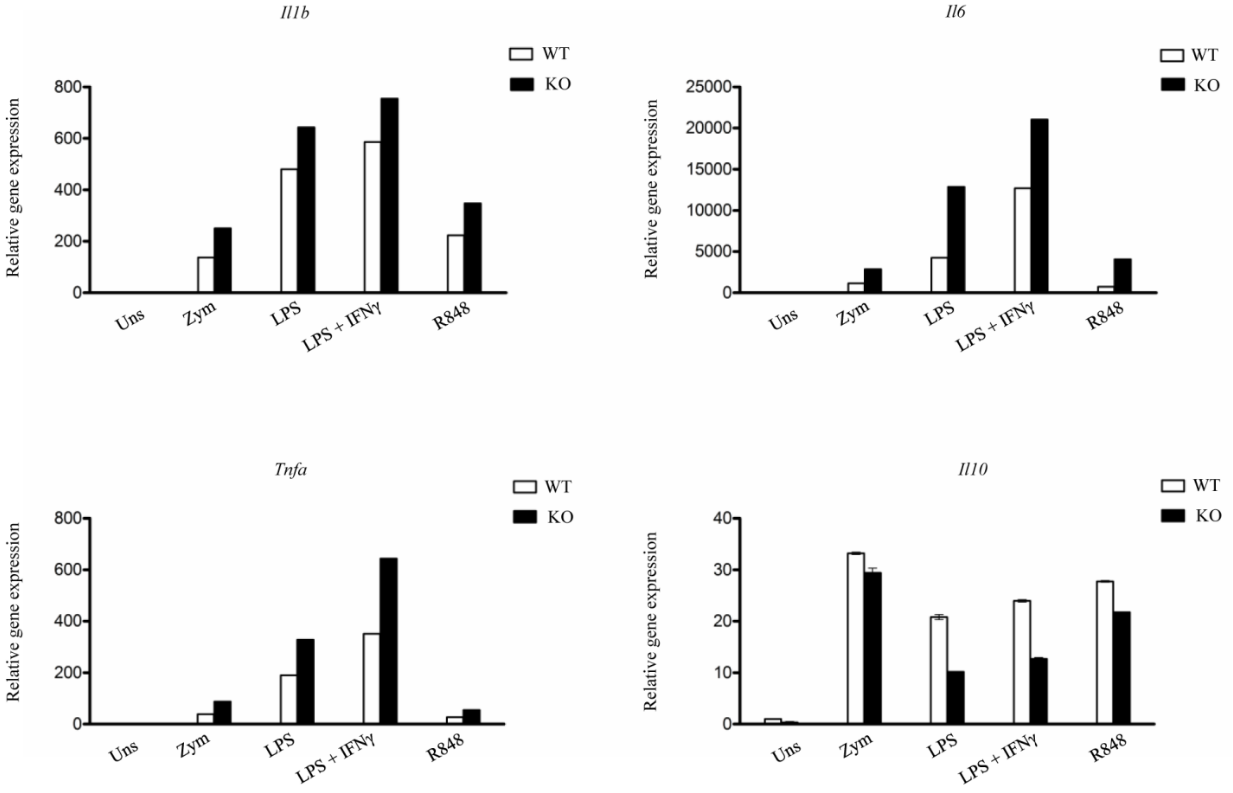

Figure S5. Cytokine mRNA expression in FURIN deficient and wild type macrophages activated with different TLR ligands. WT and FURIN KO peritoneal macrophages were left unstimulated or were stimulated for 4h with TLR ligands (Zymosan: 10 µg/ml, LPS: 1 µg/ml, R848: 1 µg/ml) and/or cytokines (IFN-γ: 20 ng/ml). Gene expression levels were determined using quantitative RT-PCR, and the relative expression in the untreated WT sample was arbitrarily set to 1. The housekeeping gene 18S was used to normalize the gene expression. One representative experiment out of three identical experiments is shown. Error bars=SEM.

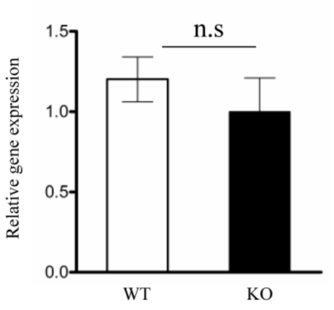

Figure S6. *Tgfb1* mRNA expression in resting wild type and FURIN deficient peritoneal macrophages. *Tgfb1* mRNA levels were determined in unstimulated WT and FURIN KO peritoneal macrophages using quantitative RT-PCR. The relative expression in the WT sample was arbitrarily set to 1. The housekeeping gene 18S was used to normalize the gene expression. (n=4/genotype) (Plots represent average ± SEM). Statistics were calculated using the two-tailed unpaired Student's t-test.

Table S1. **Sequences of the primers used for the RT-PCR.**

Primers for the RT-PCR analyses were designed using the Primer3Plus software (http://www.bioinformatics.nl/cgi-bin/primer3plus/primer3plus.cgi) and the Ensembl database (http://www.ensembl.org).

Gene	Forward	Reverse
Il1b	5'-CGT GGA CCT TCC AGG ATG AG-3'	5'-CAT CTC GGA GCC TGT AGT GC-3'
Tnfa	5'-CTT CTG TCT ACT GAA CTT CGG G-3'	5'-CAG GCT TGT CAC TCG AAT TTT G-3'
Il6	5'- TGT GCA ATG GCA ATT CTG AT- 3'	5'- CTC TGA AGG ACT CTG GCT TTG- 3'
Nos2	5'-GGG CAG TGG AGA GAT TTT GC-3'	5'-CCA GAG GGG TAG GCT TGT CT-3'
Il10	5'-GCC CAG AAA TCA AGG AGC AT-3'	5'-TGT AGA CAC CTT GGT CTT GGA G-3'
Arg1	5'-AAG AAT GGA AGA GTC AGT GTG G-3'	5'-GGG AGT GTT GAT GTC AGT GTG-3'
Pcsk1	5'-GTA CAC ATC CTA CAA TAC AGT CCA G-3'	5'-TCC CTT CTA CCC TCC ACA TT-3'
Pcsk2	5'-AGG TGT GCA GGA GAA GTT TC-3'	5'-GTC TGT CAT AAA GGG CTG GTC-3'
Furin	5'-CAG AAG CAT GGC TTC CAC AAC-3'	5'-TGT CAC TGC TCT GTG CCA GAA-3'
Pcsk4	5'-TCT TGG ACG ATG GCA TTG AG-3'	5'-TTC CAT GTC GGT TCT CAT CG-3'
Pcsk5	5'-CGC TTT CAA CGC CAA GAT TG-3'	5'-AGT CTT GCC ATC GTC ATC TG-3'
Pcsk6	5'-CTA AAC AAG CTT TCG AGT ATG GC-3'	5'-TGG TGT AGA TGC TGT TGG TG-3'
Pcsk7	5'-TTC TGT GCA GTG GGT GTG-3'	5'-CTG TCA GTA AGT GGT CCA TCC-3'
Tgfb1	5'-CCTGAGTGGCTGTCTTTTGA-3'	5'-CGTGGAGTTTGTTATCTTTGCTG-3'
18s	5'-GTG ATC CCT GAG AAG TTC CAG-3'	5'-TCG ATG TCT GCT TTC CTC AAC-3'
